# Validation of an Optogenetic Approach to the Study of Olfactory Behavior in the T-Maze of *Drosophila melanogaster* Adults

**DOI:** 10.3390/insects13080662

**Published:** 2022-07-22

**Authors:** Ruth Coya, Fernando Martin, Laura Calvin-Cejudo, Carolina Gomez-Diaz, Esther Alcorta

**Affiliations:** Department of Functional Biology, Faculty of Medicine, University of Oviedo, 33006 Oviedo, Spain; ruthct95@gmail.com (R.C.); calvinlaura@uniovi.es (L.C.-C.); gomezdiazcarolina@uniovi.es (C.G.-D.); ealcorta@uniovi.es (E.A.)

**Keywords:** behavior, *Drosophila*, olfaction, optogenetics

## Abstract

**Simple Summary:**

The fruit fly (*Drosophila melanogaster*) has been used as a model organism to study the olfactory system of insects thanks to the wide range of genetic tools available in this species. Among these tools, optogenetics allows the immediate alteration of the functioning of certain cells with light by the targeted expression of light receptor proteins in these cells. Thus, by successively expressing these receptors in different elements of the behavioral circuit, it is possible to evaluate their effect on the final behavior of the organism. However, the use of optogenetics to dissect the receptor elements of adult olfactory behavior presents a challenge because most odorants elicit gradual attraction or avoidance depending on their concentration, complicating the representative substitution of odor by light. In this work, we explore a dual excitation model in which the subject responds to various odorant concentrations while the olfactory receptor neurons are activated by light. The dose–response curve in these flies remains odorant concentration dependent, but with reduced sensitivity compared to olfactory stimulation alone. The existence of an effect associated with each of the two stimuli, odor and light, allows us to explore the quantitative contribution of the receptor elements to olfactory behavior also by optogenetics.

**Abstract:**

Optogenetics enables the alteration of neural activity using genetically targeted expression of light activated proteins for studying behavioral circuits in several species including *Drosophila*. The main idea behind this approach is to replace the native behavioral stimulus by the light-induced electrical activation of different points of the circuit. Therefore, its effects on subsequent steps of the circuit or on the final behavior can be analyzed. However, the use of optogenetics to dissect the receptor elements of the adult olfactory behavior presents a challenge due to one additional factor: Most odorants elicit attraction or avoidance depending on their concentration; this complicates the representative replacement of odor activation of olfactory sensory neurons (OSNs) by light. Here, we explore a dual excitation model where the subject is responding to odors while the OSNs are optogenetically activated. Thereby, we can assess if and how the olfactory behavior is modified. We measure the effects of light excitation on the response to several odorant concentrations. The dose-response curve of these flies still depends on odor concentration but with reduced sensitivity compared to olfactory stimulation alone. These results are consistent with behavioral tests performed with a background odor and suggest an additive effect of light and odor excitation on OSNs.

## 1. Introduction

The nervous system of animals comprises various classes of neurons interconnected with each other and forming functional circuits that control behavior and other brain functions. Deciphering how neuronal circuits cooperatively function to produce behaviors is an extremely complex task due to the enormous number of neurons and synaptic connections involved. For this reason, the study of behavioral circuits requires their precise mapping and the ability to manipulate neuronal activity in order to determine the role of each neuron and its synapses network in behavior.

Traditionally, anatomical and electrophysiological studies have been used to map neural circuits in both invertebrates and vertebrates. However, some of these invasive techniques might be unsuitable for manipulating behavioral circuits and, at the same time, observe the behavioral changes produced [1].

In 2005, optogenetics, a new non-invasive technique, was developed to alter neural activity using the genetically targeted expression of light-activated proteins, particularly ion channels and pumps [2,3]. Shortly after that, optogenetics was used to investigate behavioral circuits in several animal species from invertebrates, such as *C. elegans* and *Drosophila*, to mammals [1]. The most commonly used optogenetic tools are currently based on several microbial opsins, mainly channelrhodopsins, which are cation channels that open upon stimulation with intense blue light and subsequently depolarizes and activates neurons [4].

Olfaction is an ideal sensory system that addresses how neural circuits transform sensory input into behavior. In many animals including insects, smell-driven behaviors are essential to find food, mate or nest and the modification of these behaviors with repellents or attractants has been proposed to control insect vectors that spread human or cattle diseases or horticultural insect pests [5,6]. Moreover, the olfactory system has a common anatomical and circuit logic across the animal kingdom [7]. *Drosophila* shares with the rest of the insects most of the architecture, physiology and other molecular elements of the olfactory reception (see for example [8,9,10]). This, together with its easy maintenance in the lab, the existing behavioral paradigms [11] and the multiple genetic tools available to trace and manipulate behavioral neural circuits [12], make *Drosophila melanogaster* an attractive model to study olfaction. One of the most used genetic tools in *Drosophila* is the Gal4-UAS binary system, based in the Gal4 and UAS elements of yeast, which allows the temporal and/or spatial control of gene expression [13]. This system is composed of two parts combined in the same fly, using the genetic crosses of two lines engineered to contain molecular inserts in the DNA. In this case, a Gal4 ‘driver’ line, where a *Drosophila* promoter drives the expression of the yeast transcriptional activator GAL4 in a specific temporal and spatial pattern, and a UAS line, in which the sequence of a gene is inserted downstream of a Gal4 upstream activation sequence (UAS) to produce the expression of the gene controlled by UAS in the cells that express Gal4 [14], are present.

Optogenetic studies of olfactory behavior have been performed in *Drosophila* larvae using the Gal4-UAS binary system to express channelrhodopsin in the olfactory sensory neurons (OSNs) that express each odorant receptor molecule [14,15,16,17]. However, using optogenetics at the adult olfactory reception level presents some additional challenges compared to larvae.

Optogenetical tools have been successfully used in adults to describe an innate olfactory avoidance response to CO_2_ in a T-maze [18], but in this study, the optogenetic activation of around 70% of the OSNs, those expressing the Orco co-receptor, failed to produce a behavior. Other studies have shown, using the tracking of individual adult flies in a narrow chamber, that optogenetics in adult OSNs has to be paired with wind to elicit a behavior, when the light activation was driven either in Orco-expressing OSNs or in OSN expressing specific Odorant Receptors (ORs) [19]. Nevertheless, a more recent study has shown that optogenetics alone only elicits a behavior with the use of a few individual ORs and questions the importance of the wind [20]. In short, not only is the triggering of a behavior by light in question, but there is no unanimity about the conditions under which optogenetic activation of receptor elements can replace the native stimulus, the odorant, giving rise to an olfactory behavioral response.

Problems with the use of optogenetic methods for behavioral analysis in adults may be due to several factors. First, the reduced penetration through the adult cuticle of the short blue-light wavelength activates channelrhodopsin, especially in free olfactory behavior paradigms that are more elusive to homogeneous illumination. This fact could explain the different results obtained when optogenetics activation was driven in Orco expressing OSNs. Thus, there is no response when the light intensity is 0.06 mW/mm^2^ [18] or 0.045 mW/mm^2^ [19], but there is attraction to light with intensities ranging between 0.095 and 1.5 mW/mm^2^ [19]. For this reason, new Channelrhodopsin variants that are more appropriate for adult fly behavior analyses were created. For example, ChannelrhodopsinXXL (ChR2XXL) shows high expression levels and long open states [21] and can even be excited by the light of a mobile phone [22]. Moreover, UAS-ReaChR and CsChrimson are activated with red light that penetrates better into the adult cuticle [23,24].

Second, in *Drosophila* adults, most odorants, unlike CO_2_, elicit attraction or avoidance depending on their concentration. Such odorants are typically sensed by various types of OSNs that activate combinations of glomeruli in the antennal lobe [25], complicating the dissection, by optogenetics, of the receptor part of the circuit that translates odor recognition into behavior.

Here, we present a novel dual approach to address this issue. Specifically, we optogenetically manipulate receptor elements of the olfactory behavior circuit while the subject is responding to the native odorant stimulus in order to assess how the olfactory behavior is modified. Our protocol allows testing the response to several odorant concentrations that elicit different levels of avoidance to understand if the optogenetic changes affect the diverse quantitative levels of native stimulation differently.

To assess olfactory behavior, we use a long-established paradigm (see for example [11,26]), the T-maze, that will allow us to integrate our findings with all the existing prior knowledge on this behavioral test. Moreover, it is simpler than the paradigms used in other optogenetic studies on chemosensory behavior in adult flies [19,20].

We targeted, using an Orco-Gal4 insert, the expression of ChannelrhodopsinXXL in OSNs expressing the Orco co-receptor to observe, in a T maze, the effects produced by the light-induced activation of 70% of olfactory sensory neurons in adult olfactory responses.

## 2. Materials and Methods

### 2.1. Fly Stocks

We use the Gal-4/UAS expression system to direct the expression of Channelrhodopsin in 70% of the OSNs, those expressing the Orco co-receptor. To obtain the experimental individuals, we cross Orco-Gal4 flies [27] (w*; P{orco-GAL4.W}11.17; TM2/TM6B, Tb^1^, Bloomington Stock Center, Bloomington, IN, USA) with a UAS line, UAS-Chr2XXL (y [1] w [1118]; PBac{y[+mDint2] w[+mC] = UAS-ChR2.XXL}VK00018, Bloomington Stock Center, Bloomington, IN, USA). This line shows higher channelrhodopsin expression, enhanced subcellular localization, high retinal affinity and prolonged open-state lifetime than standard channelrhodopsin [21].

To obtain control individuals, we cross the previously mentioned UAS stock with wild-type flies (Canton-S, Bloomington Stock Center, Bloomington, IN, USA).

The crosses are made and maintained in 220 c.c. bottles with standard yeast/sucrose medium. The medium is supplemented with all-trans-retinal 300 µM (Sigma Aldrich, Steiheim, Germany) as it is the photo-switchable component of functional Channelrhodopsin [4]. Flies are cultured in a thermo-regulated chamber at 25 ± 1 °C in darkness.

### 2.2. Immunostaining

To show the OSNs that express Orco in the third antennal segment, the following fly lines were used: orco-LexA (kindly provided by Benton, R., University of Lausanne, Switzerland) and lexAop-mCD8: GFP (w*; P{y^+t7.7^ w^+mC^ = 13XLexAop2-mCD8::GFP}attP40/CyO, Bloomington Stock Center, Bloomington, IN, USA). Rabbit anti-GFP primary antibody (1:5000; Invitrogen, Waltham, MA, USA) and Alexa 488 anti-rabbit secondary antibody (1:1000; Invitrogen, Waltham, MA, USA) were used to performed an immunofluorescence on antennal cryosections in the lexAop-mCD8: GFP;orco-LexA genotype as previously described [28].

### 2.3. Behavioral Assay

The offspring of the crosses is anesthetized with CO_2_ and sorted under dim red light, outside the visible spectrum for *Drosophila*. Two-day-old females are selected and kept in the dark in groups of 20 in 25 c.c. tubes feeding only on water for 24 h prior to the behavioral test.

**Figure 1:**
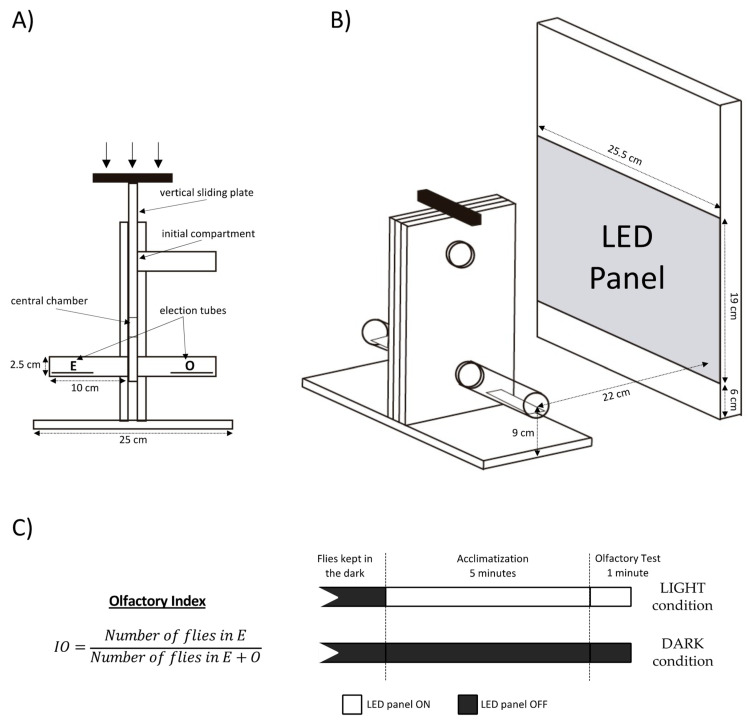
Dual-light odorant-excitation olfactory setup. (**A**) Schematic representation of the T-maze. (**B**) Relative positions of the T-maze and the LED panel for the optogenetic tests. (**C**) Olfactory Index formula (**left**) and illumination conditions used in the dose–response curve of ethyl acetate (**right**).

We perform a double-choice fast olfactory preference test, the T-maze, (Figure 1A) using a previously described protocol [29]. In summary, for each replicate test groups of 20 flies are introduced in the initial compartment and forced to the central chamber of a vertical sliding plate, the elevator. Once the plate slides to the bottom position, they can choose between two tubes for one minute. One tube contains a filter paper soaked with 0.5 mL of ethyl acetate diluted in paraffin oil (E), while the other contains only 0.5 mL of the solvent (O). Both ethyl acetate and paraffin oil were supplied by MERCK (Darmstadt, Germany). Six odorant concentrations (vol/vol) are tested (10^−3.5^, 10^−3^, 10^−2.5^, 10^−2^, 10^−1.5^ and 10^−1^) and 20 replicates of 20 flies each were performed for each concentration (6), genotype (2) and lighting condition (2).

All replicate tests are carried out at constant temperature and relative humidity (24 ± 1 °C and 40–60% RH) between 4 p.m. and 7 p.m.

To avoid cross effects on the response due to lateral preference, geotropic or phototropic effects, the stimulus is placed alternatively on the right and left side of the maze. In addition, the control and experimental groups are also alternated to avoid a possible “time effect” throughout the testing period.

After the test, the tubes are disconnected, capped and the flies are counted. Replicates in which less than 5 individuals reach the end of the maze are discarded.

An olfactory index (IO) is calculated as the number of flies in the stimulus tube (E) divided by the total number of flies reaching the end of the maze at either end (E+O) (Figure 1C left). IO values range from 0 (maximum repulsion) to 1 (maximum attraction), with the indifference value at 0.5.

A comparison is made among the IO of the experimental and control hybrids, using a one-way ANOVA statistical test for each odorant concentration followed by a Bonferroni post hoc analysis for comparison among multiple groups using the IBM SPSS Statistics software versions 26 and 27 for Macintosh.

### 2.4. Optogenetics

For channelrhodopsin activation, we use a high-intensity white LED spotlight (Alverlamp LSPR0200 W 40, Valencia, Spain) of 200 W, 4000 K and 18,000 lumens. This LED panel is located 22 cm in parallel to the T-maze tubes, as shown in Figure 1B, producing a constant luminance of 78,612 lux, which corresponds to a 0.43 mW/mm^2^ irradiance, within the range that evokes a behavioral response according to previous reports [19].

To obtain the dose–response curve relative to ethyl acetate, four sets of flies are tested according to genotype and illumination condition (Figure 1C right). The same flies are never tested twice. Under the LIGHT condition, flies are subjected to 5 min of light before being connected to the maze and then to another minute of light inside the T-maze during the odor preference test. Under the DARK condition, individuals are kept 5 + 1 min under dim red light before being connected to the maze and during the test. Subjecting the flies 5 min before the test to the same light condition as during the test adds some stability to the response.

**Figure 2:**
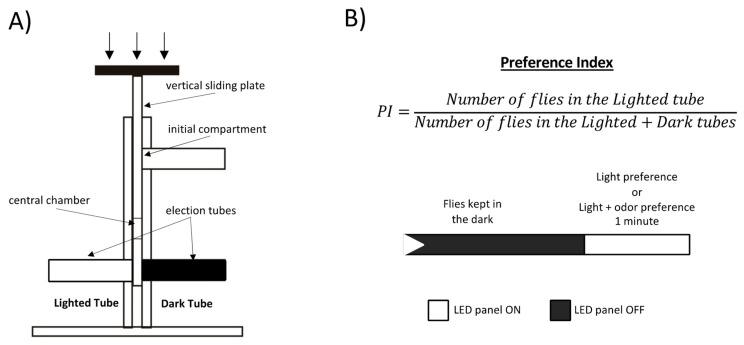
Light attraction setup. (**A**) Schematic representation of the modified T-maze. (**B**) Preference Index formula (**top**) and illumination conditions used in the light attraction test (**bottom**).

In order to explain the results obtained in the dose–response curve we used the same paradigm, protocols and environmental conditions but with one of the election tubes painted in black (Dark Tube) (Figure 2A) to see the effect of the light alone without odor for both experimental (orco-Gal4/UAS-ChR2XXL) and control (CantonS/UAS-ChR2XXL) genotypes, and the effect of light plus odor in the control genotype (CantonS/UAS-ChR2XXL) with the transparent tube (Lighted Tube) containing a filter paper soaked with 0.5 mL of ethyl acetate 10^−2.5^ or 10^−2^ dilutions in paraffin oil. About 20 replicates of 20 flies each are performed for each genotype and condition. Flies are kept in the dark and once the plate slides to the bottom position, the LED panel is lit on and they can choose between Lighted or Dark Tubes for one minute (Figure 2B bottom). A preference index is calculated as the number of flies in the Lighted Tube divided by the total number of flies reaching the end of the maze at either end, Lighted or Dark Tube (Figure 2B top). These index values range from 0 (maximum repulsion) to 1 (maximum attraction), with the indifference value at 0.5.

## 3. Results and Discussion

**Figure 3:**
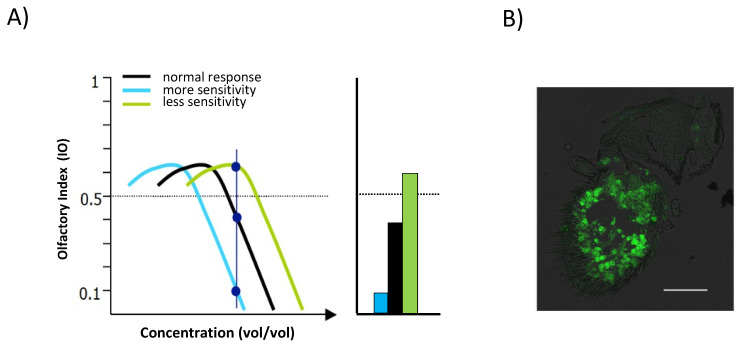
Representative dose–response curve in the T-maze. (**A**) Representation of the typical dose-response curve in the T-maze in response to increasing concentrations of odor (**left**). Testing a single odorant concentration, corresponding to the vertical blue line in the dose–response curve, in the repellent region for normal flies (black) allows detecting changes in sensitivity in both directions, either less sensitive flies (green) or more sensitive flies (blue). IO mean values are represented in the histogram (**right**). Note that in this case the higher IO values correspond to diminished sensitivity and the lower values to increased sensitivity. (**B**) Representative immunostaining of the main olfactory organ of *Drosophila* where the OSNs expressing the Orco co-receptor cover the entire surface of the third antennal segment. Green Fluorescent Protein (GFP) directed expression in these OSNs is shown in green. Scale bar, 30 μm.

The typical dose–response curve obtained in a T maze in response to increasing concentrations of odor is represented in Figure 3A, which compares flies with normal sensitivity (in black) with flies with increased (in blue) or decreased (in green) sensitivity. It should be noted that this paradigm shows both qualitative information with an attractive and an aversive region of concentrations and some quantitative information of gradual response, especially evident in the aversive area, where increasing concentrations of odorant evoke increasing aversive behaviors.

As the same olfactory response value can be obtained with two different concentrations in the attractive region but not in the repellent region, we have chosen, for our experiments, mainly those concentrations that evoke a repellent response, as they allow us to study quantitative differences in both directions: increasing and decreasing olfactory sensitivity (Figure 3A). We relate sensitivity in the experimental line to the control line values. Thus, we define a line as more sensitive than normal if we need a higher odorant concentration to obtain the same IO value in the control line, and contrarily, it is less sensitive than normal if we need a lower odorant concentration to obtain the same IO value in the control line.

The selective expression of channelrhodopsin in the experimental group affects those OSNs that express the Orco co-receptor located at the third antennal segment (Figure 3B) and the maxillary palp (not shown) of *Drosophila*.

**Figure 4:**
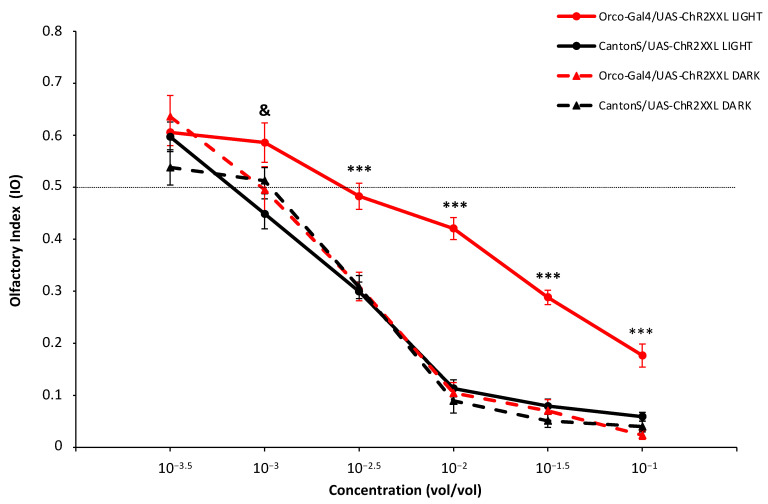
Olfactory behavior with optogenetic activation of Orco-expressing Olfactory Sensory Neurons (OSNs). Dose–response curve of olfactory responses to ethyl acetate in four fly genotypes. Each point shows the average Olfactory Index (IO) ± SEM for each concentration, genotype and light condition. At the 10^−3^ concentration, only differences between Orco-Gal4/UAS-Chr2XXL LIGHT and CantonS/UAS-ChR2XXL LIGHT became statistically significant (& = *p* < 0.05). For the 10^−2.5^ to 10^−1^ concentrations, highly significant differences (*** = *p* < 0.001) appeared between Orco-Gal4/UAS-Chr2XXL LIGHT and the other 3 classes, which were equal to each other.

A dose–response curve is performed in response to six odorant concentrations of ethyl acetate in both the experimental (expressing channelrhodopsin) and control flies in the two lighting conditions, as shown in Figure 4. For the four higher concentrations, there are highly significant differences among groups (10^−2.5^ F_3,76_ = 14.145 *** *p* = 2.05 × 10^−7^; 10^−2^ F_3,76_ = 60.371 ***, *p* = 4.56 × 10^−20^; 10^−1.5^ F_3,76_ = 50.504 ***, *p* = 4.63 × 10^−18^; 10^−^^1^ F_3,76_ = 26.148 ***, *p* = 1.01 × 10^−11^; *** = *p* < 0.001).

Subsequent post hoc comparisons were carried out to uncover differences between particular groups. The experimental individuals subjected to intense light (Orco-Gal4/UAS-CHR2XXL LIGHT) present significantly less repellent responses (*** *p* < 0.001) than the other three groups (CantonS/UAS-CHR2XXL LIGHT, Orco-Gal4/UAS-CHR2XXL DARK and CantonS/UAS-CHR2XXL DARK) (Figure 4). There are no differences between experimental and the other three classes for ethyl acetate 10^−3.5^ (F_3,76_ = 1.627, *p* = 0.19 n.s.), but for the 10^−3^ concentration (F_3,70_ = 2.733 *, *p* = 0.05, * = *p* ≤ 0.05), there is a significant difference between Orco-Gal4/UAS-CHR2XXL and CantonS/UAS-CHR2XXL under the LIGHT condition when a post hoc mean comparison is performed (* *p* = 0.039) but not between the Orco-Gal4/UAS-CHR2XXL in the LIGHT and DARK conditions. Therefore, we cannot assign an additional effect to the opening of channelrhodopsin at this odorant concentration. No differences have been found neither between the control group (CantonS/UAS-CHR2XXL) in which there is no expression of channelrhodopsin, either in the DARK or LIGHT conditions (Figure 4).

Therefore, in our tests optogenetic as well as odorant stimulation are producing an effect in the final behavioral response in the experimental flies (Orco-Gal4/UAS-CHR2XXL) in the LIGHT condition, where the channelrhodopsin expressed in the OSNs was activated. Moreover, increasingly high odorant concentrations of ethyl acetate produce quantitatively increasing repellent responses in the T-maze in all cases as has been extensively reported (see for example, [30]). However, the addition of optogenetic activation to the OSNs modifies behavior quantitatively in a manner that can be directly related to the excitation produced at the receptor level. Thus, our results resemble those cases where response to odorant pulses is tested under a background odor. Only those concentrations that are higher to the background are able to evoke a response and, in fact, this can be already seen at electrophysiological measurements [31,32]. In fact, it has been previously proposed that flies’ olfactory response is not determined by absolute OSN activity, but by activity relative to the background [33].

In our case, flies with dual activation have to choose in the T-maze between the equivalent to a certain concentration of background odor (optogenetic activation only) in one arm and with odor + background odor in the other arm. Thereby, the repulsion or attraction by the odor branch (E) is expected to be equal to the repulsion or attraction of the light-only branch (O) when the real and virtual odor concentrations approach each other, in our case around 10^−2.5^ (vol/vol). At this point, in fact, the behavioral attraction or repulsion between the two branches disappeared and induced an indifferent response in the T-maze.

**Figure 5:**
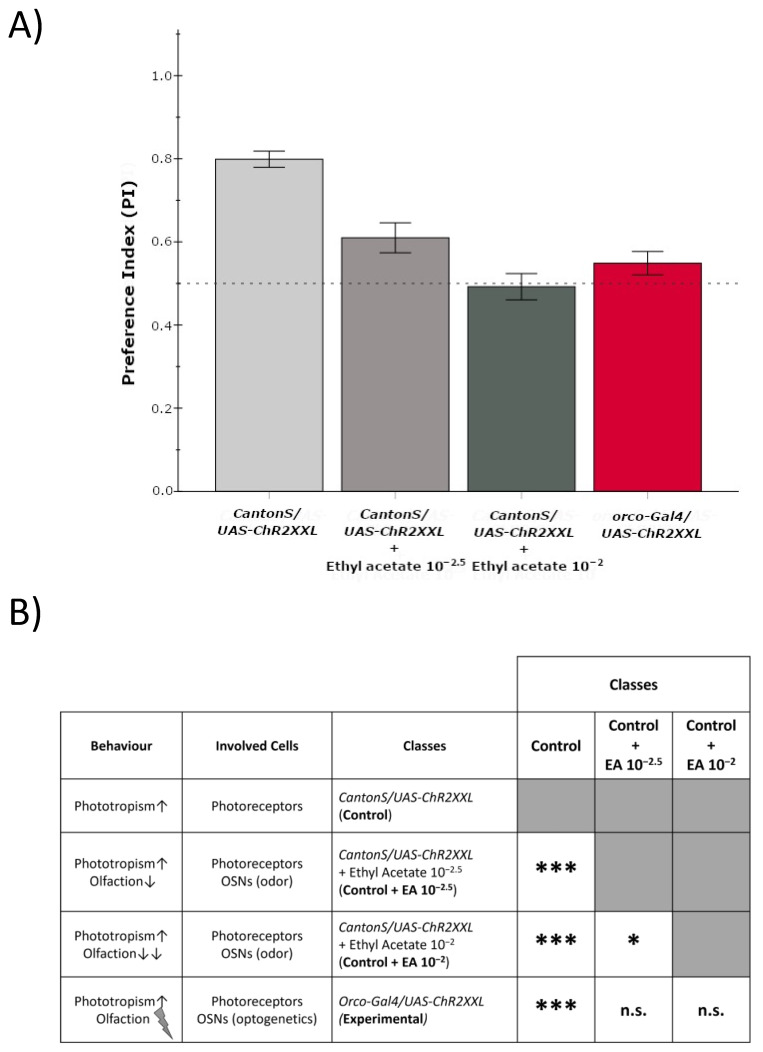
Phototropism vs. Olfactory Response. (**A**) Response to light of experimental individuals (Orco-Gal4/UAS-Chr2XXL) with optogenetic activation of Orco-expressing Olfactory Sensory Neurons (OSNs) compared with control individuals (CantonS/UAS-ChR2XXL) responding to light or a combination of light and odor (Ethyl Acetate 10^−2.5^ and 10^−2^). Bars represent Mean PI ± SEM. (**B**) Table of significant differences. The behavior and cells involved in the response of each of the four classes compared are presented along the significant differences found among the classes. (* = *p* < 0.05; *** = *p* < 0.001; n.s. = not significant differences). The white squares represent all possible two-by-two comparisons. The gray squares correspond to nonsense or repeated comparisons.

To test the hypothesis of the dual activation of OSNs for explaining our results, we tried to dissect the effects of light and odorant excitation in our experiment. Thus, we modified the T maze, as shown in Figure 2A, by darkening one of the choice tubes to measure the response to light only. As can be seen in Figure 5, there are significant differences between the tested classes (ANOVA F_3,73_ = 21.680 ***, *p* = 3.82 × 10^−10^, *** = *p* < 0.001) and the post hoc comparison among groups that are significantly different are shown in the table of Figure 5B. The control individuals (CantonS/UAS-CHR2XXL) are attracted to the Lighted Tube (PI = 0.799 ± 0.019). Meanwhile, the experimental individuals (Orco-Gal4/UAS-CHR2XXL), which undergo the optogenetic excitation of most OSNs, are practically indifferent to light (PI = 0.549 ± 0.028). There are significant differences between both classes (*p* = 2.65 × 10^−7^ ***). Given that neither of the two types of flies are blind, as occurring in other studies [18,19], what we would be observing is that there is only a positive phototropism due to the visual reception system in the controls, while this positive phototropism would be practically canceled by the aversive effect produced by the optogenetic activation of OSNs in the experimental flies.

The same effect can be simulated by a combination of light and odor in the control flies (CantonS/UAS-CHR2XXL) introducing an odorous stimulus in the Lighted Tube (Figure 5A). When a filter paper containing ethyl acetate 10^−2.5^ is added, the aversive effect of the odorant by itself (see Figure 4 for the control flies as well as the experimental flies in the dark, when the OSNs are not activated by light) counteracts the light attraction effect by decreasing the PI to 0.610 ± 0.036. As the odor concentration increases (ethyl acetate 10^−2^), so does the repulsion for the odor, and the PI drops even further, down to 0.492 ± 0.032. Both values are significantly different from the response of the control flies to the light alone (*p* = 1 × 10^−4^ *** and *p* = 4.92 × 10^−10^ ***, respectively; *** = *p* < 0.001), but they do not differ significantly to the response of the experimental flies (Orco-Gal4/UAS-CHR2XXL) to light alone (*p* = 0.9036 n.s. and *p* = 1.0 n.s., respectively) (Figure 5B). Again, these results place the approximate equivalence of the optogenetic activation of OSNs in our conditions around the 10^−2.5^ concentration of ethyl acetate. In previous studies the optogenetic activation of Orco-expressing OSNs produced attraction in all but the smaller light intensities used in adult males [19,20] and starved females [20], but the genotypes, behavioral paradigms and light sources are not the same as the ones used in this work, and, probably, in their conditions, higher light intensities could have also produced repulsive behaviors.

These results reinforce the idea that this dual approach is suitable for investigating the quantitative contribution of olfactory receptor elements to the olfactory behavior in a typical T-maze paradigm with homogeneous intense LED illumination. More precisely, a significant role of a certain receptor element in behavior could be identified as a quantitative change of olfactory preference to an odorant concentration.

This approach could also be valuable for investigating olfactory behavior to repellents or attractants that can be useful as control strategies for insects that cause human vector-borne diseases or affect livestock or crops. In fact, *Drosophila* has been used to study the olfactory behavior of some repellents that could be used to control harmful insects [34]. For some years now, progress has been made in the study of the olfactory system of several insect vectors of human diseases (see for example [35]). Moreover, new transcriptomic analysis techniques have made it possible to study the olfactory system of many other pest insects (see for example [36,37]). These types of studies could allow the discovery of species-specific components of their olfactory systems, such as odorant receptors or odorant-binding proteins (OBPs) that could be exploited to manipulate olfactory behavior in specific insects. This is important, because other olfactory receptor elements such as the coreceptor Orco could be a useful target for manipulating olfactory behavior as it is essential for many OSNs as shown in this work. On the other hand, its phylogenetic and function conservation among insects [38,39,40,41] means that manipulating Orco to control pest insects could also affect beneficial insects. To use our optogenetic approach in other insects, it should be necessary to develop and use the binary Gal4/UAS system in other insects besides *Drosophila* [42]; however, in fact, this has already been carried out in the mosquito *Aedes aegypti* to study olfactory receptors [43] and to manipulate a pheromone receptor in the silk moth by optogenetics [44]. The progressive development of targeted expression systems will increase the possibilities of extending studies of the olfactory system for other insects.

## Data Availability

The data presented in this study are available upon request from the corresponding author. The data are not publicly available due to privacy reasons.
